# Transdiagnostic early warning score for psychiatric hospitalisation: development and evaluation of a prediction model

**DOI:** 10.1136/bmjment-2025-301622

**Published:** 2025-07-17

**Authors:** Maxime Taquet, Seena Fazel, A John Rush

**Affiliations:** 1 Department of Psychiatry, University of Oxford, Oxford, UK; 2 Oxford Health NHS Foundation Trust, Oxford, UK; 3 Medical School, Duke-National University of Singapore, Singapore

**Keywords:** Anxiety disorders, Depression & mood disorders, Personality disorders, Schizophrenia & psychotic disorders, Mental Health Services

## Abstract

**Background:**

The lack of an early warning score for psychiatric hospitalisation means that the decision to initiate preventative interventions is based solely on clinical judgement, which is prone to bias.

**Objective:**

The objective is to develop and externally validate a transdiagnostic score that predicts psychiatric hospitalisation.

**Methods:**

In this retrospective cohort study using deidentified electronic health records from 20 healthcare organisations in the NeuroBlu Data, we identified all patients with any of seven major psychiatric disorders with at least five Clinical Global Impressions of Severity and five Global Assessment of Functioning measured over a period of 6 consecutive months before any hospitalisation. From these measurements, metrics of clinical severity and instability and functional severity and instability were derived and incorporated into a score predicting the 6-month risk of incident psychiatric hospitalisation. Discrimination and calibration of this score were validated in an external sample. The transdiagnostic validity of the score was evaluated and its performance was compared between white and non-white people.

**Findings:**

Altogether, 37 049 individuals (531 incident hospitalisations) were included. The predictive model showed good discrimination in the training (optimism-adjusted c-index: 0.74, 95% CI 0.72 to 0.76) and external validation (c-index: 0.80, 95% CI 0.78 to 0.82) samples, with adequate calibration. Discrimination improved with adjustment for organisation-level hospitalisation rates (c-index: 0.80, 95% CI 0.78 to 0.82 and 0.84, 95% CI 0.82 to 0.86 in the derivation and validation samples). Good discrimination was also achieved for each diagnostic category (c-index: 0.71–0.82 and 0.64–0.75 with/without adjustment for organisation-level hospitalisation rates, respectively). There was no significant difference in model performance between white and non-white people.

**Discussion:**

A transdiagnostic early warning system based on simple longitudinal measurements can reliably and robustly predict psychiatric hospitalisation. It will help target preventative interventions to individuals most at risk.

**WHAT IS ALREADY KNOWN ON THIS TOPIC**

- Predicting the risk of hospitalisation across psychiatric disorders would enable more effective targeting of preventative strategies. However, existing predictive systems are limited to specific diagnoses or clinical scenarios and primarily focus on forecasting readmissions after an initial hospitalisation, reducing their applicability.

**WHAT THIS STUDY ADDS**

- This study introduces and externally validates a clinical prediction model that accurately predicts the 6-month risk of psychiatric hospitalisation across seven major psychiatric diagnoses.

**HOW THIS STUDY MIGHT AFFECT RESEARCH, PRACTICE OR POLICY**

- This score can help target preventive interventions to those at high risk, reducing the burden of psychiatric hospitalisation on individuals and their families while also helping service provision planning.

## Background

 Early warning scores are ubiquitous in general hospitals, measured hundreds of millions of times each year.^1^ These scores can predict deterioration in physical health from a few vital signs. Coupled with effective interventions, they can substantially improve patient outcomes.^2^

No such early warning score is used to predict the worsening of mental health conditions. Consequently, identifying patients most at risk of deterioration is based on the clinician’s intuition, which is prone to bias.^3^ This might disproportionately affect people from ethnic minorities who face a higher risk of involuntary admission.^4^ Underestimating the risk of deterioration can lead to missed opportunities for early interventions and dire consequences for patients, whereas overestimating the risk leads to wasted resources. A key indicator of deterioration in mental health is the need for hospitalisation—typically reserved for the most severely unwell patients. An early warning score for patients with mental health thus ought to be able to help clinicians predict psychiatric hospitalisation.

Psychiatric hospitalisation is a scarce resource and is associated with high cost,^5^ stigma^6^ and disrupted educational and professional development.^7^ Intensive interventions, such as involvement of a crisis resolution team, are implemented to prevent admission when possible.^8^ Predicting the risk of hospitalisation would improve the targeting of these interventions. This question has interested researchers for over 50 years,^9^ and several studies have identified factors that predict psychiatric hospitalisation. However, these predictors have been limited to specific diagnoses^10^,^13^ and clinical scenarios (eg, patients in crisis^14^). Very few prediction models have been built on them,^13^,^15^ and all were limited to data from one or two organisations, precluding assessment of the generalisability of the models.

Recently, clinical instability (the visit-to-visit fluctuation in clinical severity) has been identified as a strong predictor of hospitalisation across psychiatric diagnoses independently of average clinical severity.^16^ Here, we combined clinical and functional *instability* with clinical and functional *severity* measurements to develop and externally validate an early warning score for psychiatric hospitalisation.

## Methods

### Study design and data source

This retrospective cohort study leveraged electronic health record (EHR)-derived, de-identified data from NeuroBlu Data, a longitudinal behavioural health real-world database comprising structured and unstructured patient-level clinical data from mental health centres across the USA spanning over 20 years. Mental health centres provide secondary psychiatric care in both inpatient and outpatient settings.

The primary data came from the sites using the MindLinc EHR system spanning 12 US states, and accessed via NeuroBlu release 23R3, described elsewhere.^17^ Only sites with fewer than 25% of their patients being hospitalised were included to exclude organisations with predominantly inpatient facilities (for which patients might receive community care in organisations not included in the network). This resulted in data from 20 different sites. Structured data included sociodemographic factors and diagnosis (ie, International Classification of Diseases (ICD)-9 and ICD-10 codes). Additionally, a longitudinal clinician-rated Clinical Global Impression of Severity (CGI-S) measuring clinical severity and a clinician-rated Global Assessment of Functioning (GAF) measuring functional ability were available (recorded between March 1999 and September 2021). These ratings were not typically used to make treatment decisions, and clinicians were not explicitly trained in them.

This study followed the Transdiagnostic (TRANSD) recommendations^18^ (when applicable) and the Transparent Reporting of a multivariable prediction model for Individual Prognosis Or Diagnosis (TRIPOD+AI) guidelines. All analyses were prespecified (but the study was not preregistered), and a statistical analytic plan is available in online supplemental material A. Patients and the public were not involved in the design and conduct of this study.

### Cohort

We included all patients with an ICD-9 or ICD-10 code for major depressive disorder (MDD), bipolar disorder (BD), generalised anxiety disorder (GAD), post-traumatic stress disorder (PTSD), schizophrenia or schizoaffective disorder (SCZ), attention deficit hyperactivity disorder (ADHD) or personality disorder (PD) as in our previous study.^16^ Diagnoses were not mutually exclusive: each diagnosis was represented with a binary variable (present vs absent) in the statistical model. Included patients had to have, within any 180 consecutive days (referred to as the ‘measurement period’) before any hospitalisation, at least five measurements of CGI-S and five measurements of GAF so that prediction occurred while these persons were outpatients. See online supplemental material B and C for further details on cohort definitions.

The cohort was separated into a derivation sample and a separate external validation sample based on the healthcare organisations that patients were seen at, with organisations that provided the most recent data used as a derivation sample (15 sites, n=30 493 patients, 409 with psychiatric hospitalisations), and organisations providing more historical data kept as external validation (5 sites, n=6556 patients, 122 with psychiatric hospitalisations; see online supplemental material D). This separation based on calendar time and healthcare organisation is preferred to a random split of the population, which is both inefficient^19^ and fails to test the transportability of the model to other populations.^20^

### Predictors

From consecutive measurements of CGI-S, two metrics were calculated: clinical severity (the average of all CGI-S within the 180 days) and clinical instability (the time-adjusted root mean square of successive differences (*t*RMSSD) of all CGI-S as in previous work^16^). The latter measures the visit-to-visit fluctuations in CGI-S. Similarly, functional severity and instability were calculated as the average and *t*RMSSD of GAF (after inverting the scale so that higher values represent more impairment). See online supplemental material E-G for details on predictors definition.

### Outcome

The primary outcome was any psychiatric hospitalisation coded using the Observational Medical Outcomes Partnership Common Data Model^17^ within 180 days after the end of the 180-day measurement period. This period was chosen as it reflects the period during which crisis resolution intervention effectively prevents hospitalisation.^8^ Patients who had no hospitalisation were censored at the time of their last visit to mental health services or at 6 months, whichever came first.

### Statistical power

No formula links differences in *t*RMSSD to statistical power. Instead, as in our previous study,^16^ we relied on an empirical framework^21^ to assess statistical power (using affective time series as proxies for CGI-S/GAF time series). With a minimum group size of 4991 (representing the smallest subgroup in our study) to reach a statistical power of 90% to detect a small effect (1% of the variance) with an alpha of 0.05, five observations per individual are enough to calculate instabilities.

### Statistical analysis

To assess whether people with at least five CGI-S and GAF measurements differ from the broader cohort of people with any number of CGI-S and GAF measurements, we compared the characteristics and outcomes between the two groups. We report the standardised mean difference (SMD) and consider an SMD≤0.1 to represent little discrepancy between cohorts.

In the derivation sample, we conducted a time-to-event analysis using the Cox proportional hazard model with each of the four predictors as independent variables alongside diagnosis, gender (as recorded in the individual’s EHR) and age. This model was chosen as it is interpretable, which is important for high-stakes decisions^22^ and requires no hyperparameter tuning. There were no data missing for age. Unknown gender was considered as a separate category, alongside the other categories. Adjustment for the probability of psychiatric hospitalisation (logit-transformed) at the organisation level was also considered to account for interorganisation differences in propensity to hospitalise patients. We refer to the model with the latter adjustment as the ‘adjusted’ model in contrast to the ‘unadjusted’ model, where this adjustment is not included. For instabilities, variable transformation (using the square root transform) was applied if it led to a lower Akaike information criterion. The generalised Schoenfeld approach tested the proportional hazard assumption^23^ and rejected it if p<0.05.

In internal validation, discrimination of the model was assessed by computing the optimism-corrected c-index (using bootstrap with 200 repetitions),^24^ as well as sensitivity, specificity, positive predictive value (PPV) and negative predictive value (NPV) for a prespecified estimated incidence of hospitalisation of 2%. CIs for all these quantities were calculated using bootstrap with 200 repetitions. Calibration was assessed by comparing the predicted and observed risks, with CIs established using bootstrap with 200 repetitions.^24^ We also report the Brier score that quantifies the accuracy of the predictions. The Brier score ranges from 0 to 1, with lower scores indicating better calibration. It represents the mean squared error of the predicted probabilities.

To assess the external validity of the predictive model, the same discrimination statistics listed above and the relation between the predicted and observed risks were calculated in the external validation sample based on the model learnt using the derivation sample. Bootstrap with 1000 repetitions was used to calculate CIs for the c-index. Wilson score CIs were calculated for the observed risks.

To assess the transdiagnostic validity of the model, receiver operating characteristics and c-indices were computed after applying the model to each sub-cohort defined based on individual diagnostic categories. To start to assess the fairness of the model, we evaluated the model separately in white and non-white people (excluding those with unknown race) and compared the c-indices between the two groups using permutation tests with 100 permutations.

All analyses were repeated with a ‘baseline’ model including only diagnosis, gender and age as predictors and a ‘clinical benchmark’ model including diagnosis, gender, age and clinical severity to mimic the information clinicians likely use to make clinical decisions. The statistical significance of the differences in c-index (ie, whether they depart from zero) between these two models (baseline and clinical benchmark) and the primary models (unadjusted and adjusted) was assessed using bootstrapping with 1000 repetitions in the external validation sample. Statistical significance was set at two-sided p values<0.05. Analyses were conducted in R V.4.0.4, using the rms package V.6.1-1. More details on statistical analyses are provided in online supplemental material H.

## Results

A total of 37 049 patients were included (mean (SD) age: 32.1 (18.7) years, 57.6% female gender, 42.4% male gender; see table 1 for baseline characteristics). The selected cohort was broadly similar to the wider population with any number of CGI-S/GAF measurements across sites (including in terms of baseline clinical and functional severity and hospitalisation incidence), but had a lower proportion of white individuals and a higher prevalence of SCZ and PD (online supplemental table 1).

**Table 1:** Characteristics of the derivation and external validation samples

	Full cohort	Derivation	External validation
Cohort size	37 049	30 493	6556
Age (years), mean (SD)	32.1 (18.7)	32.2 (18.7)	31.5 (18.6)
Gender, n (%)			
Female	21 335 (57.6)	17 495 (57.4)	3840 (58.6)
Male	15 706 (42.4)	12 991 (42.6)	2715 (41.4)
Unknown	8 (0.022)	7 (0.023)	1 (0.015)
Race, n (%)			
American Indian or Alaska Native	146 (0.39)	131 (0.43)	15 (0.23)
Asian	302 (0.82)	242 (0.79)	60 (0.92)
Black or African American	7183 (19.4)	5929 (19.4)	1254 (19.1)
Native Hawaiian or other Pacific Islander	43 (0.12)	41 (0.13)	2 (0.031)
White	19 551 (52.8)	15 811 (51.9)	3740 (57.0)
Other/unknown	9824 (26.5)	8339 (27.3)	1485 (22.7)
Diagnosis, n (%)			
MDD	13 503 (36.4)	10 460 (34.3)	3043 (46.4)
BD	8271 (22.3)	7168 (23.5)	1103 (16.8)
GAD	4991 (13.5)	3722 (12.2)	1269 (19.4)
PTSD	8036 (21.7)	6799 (22.3)	1237 (18.9)
SCZ	4737 (12.8)	4410 (14.5)	327 (4.99)
ADHD	8737 (23.6)	6987 (22.9)	1750 (26.7)
PD	5236 (14.1)	4281 (14.0)	955 (14.6)
Predictors			
Clinical severity, median (IQR)	4.02 (3.68–4.80)	4.08 (3.75–4.86)	4.00 (3.43–4.46)
Functional severity, median (IQR)	52.6 (45.7–59.0)	51.0 (44.8–57.0)	60.5 (54.2–65.8)
Clinical instability, median (IQR)	0.049 (0.00–0.19)	0.054 (0.00–0.22)	0.038 (0.01–0.094)
Functional instability, median (IQR)	0.24 (0.083–0.77)	0.24 (0.076–0.81)	0.27 (0.12–0.63)
Follow-up (days), median (IQR)	180 (180–180)	180 (180–180)	180 (180–180)
Admissions, n (%)	531 (1.43)	409 (1.34)	122 (1.86)

Comparison of the characteristics with those of the wider population can be found in online supplemental table 1.

ADHD, attention-deficit hyperactivity disorder; BD, bipolar disorder; GAD, generalised anxiety disorder; MDD, major depressive disorder; PD, personality disorder; PTSD, post-traumatic stress disorder; SCZ, schizophrenia or schizoaffective disorder.

In the derivation sample, within the model unadjusted for the organisation-wide probability of hospitalisation, all four predictors were significantly associated with subsequent psychiatric hospitalisation within the next 6 months after the measurement period: adjusted HR of 1.49 (95% CI 1.34 to 1.65, p<0.0001) for every SD increase in functional severity, 1.20 (95% CI 1.11 to 1.29, p<0.0001) for functional instability, 1.15 (95% CI 1.04 to 1.27, p=0.0056) for clinical severity and 1.13 (95% CI 1.03 to 1.24, p=0.0066) for clinical instability. Other predictors significantly associated with psychiatric hospitalisation included diagnoses of BD (HR 1.53, 95% CI 1.23 to 1.91, p=0.0001), SCZ (HR 1.92, 95% CI 1.50 to 2.45, p<0.0001) and ADHD (HR 0.47, 95% CI 0.32 to 0.69, p=0.0001). In the model adjusted for the organisation-wide probability of hospitalisation, the same predictors were significantly associated with psychiatric hospitalisation except for clinical severity (no longer significant) and male gender (which became significantly associated with an increased risk of hospitalisation). All coefficients and their p values for both the adjusted and unadjusted models are provided in online supplemental tables 2 and 3. There was no evidence of non-proportional hazards (χ^2^: 15.39, p=0.35 for the unadjusted model and 15.97, p=0.38 for the model adjusted for the organisation-wide probability of hospitalisation), so that the HR derived from the proportional hazard model can be used.

**Figure 1:**
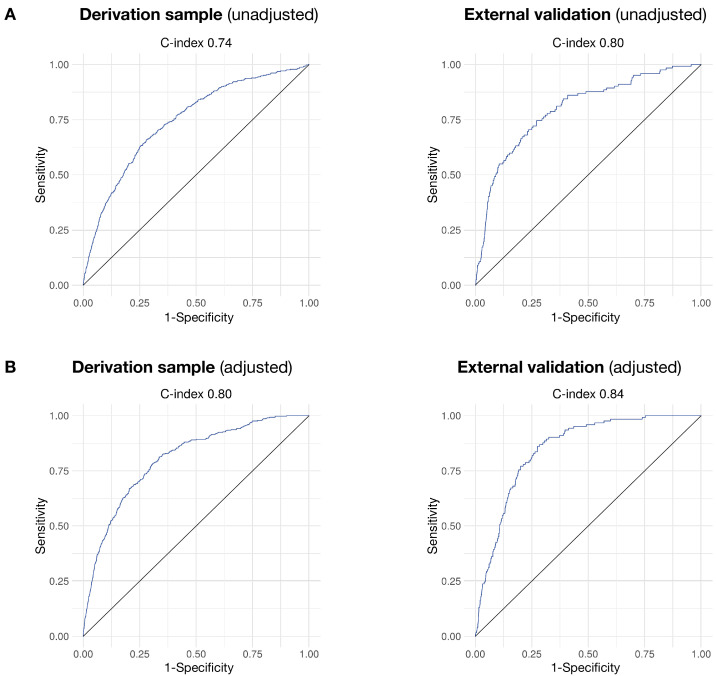
Receiver operating characteristics for the prediction of 6-month hospitalisation using (A) the unadjusted model and (B) the model adjusted for interorganisation differences in hospitalisation propensity.

**Figure 2:**
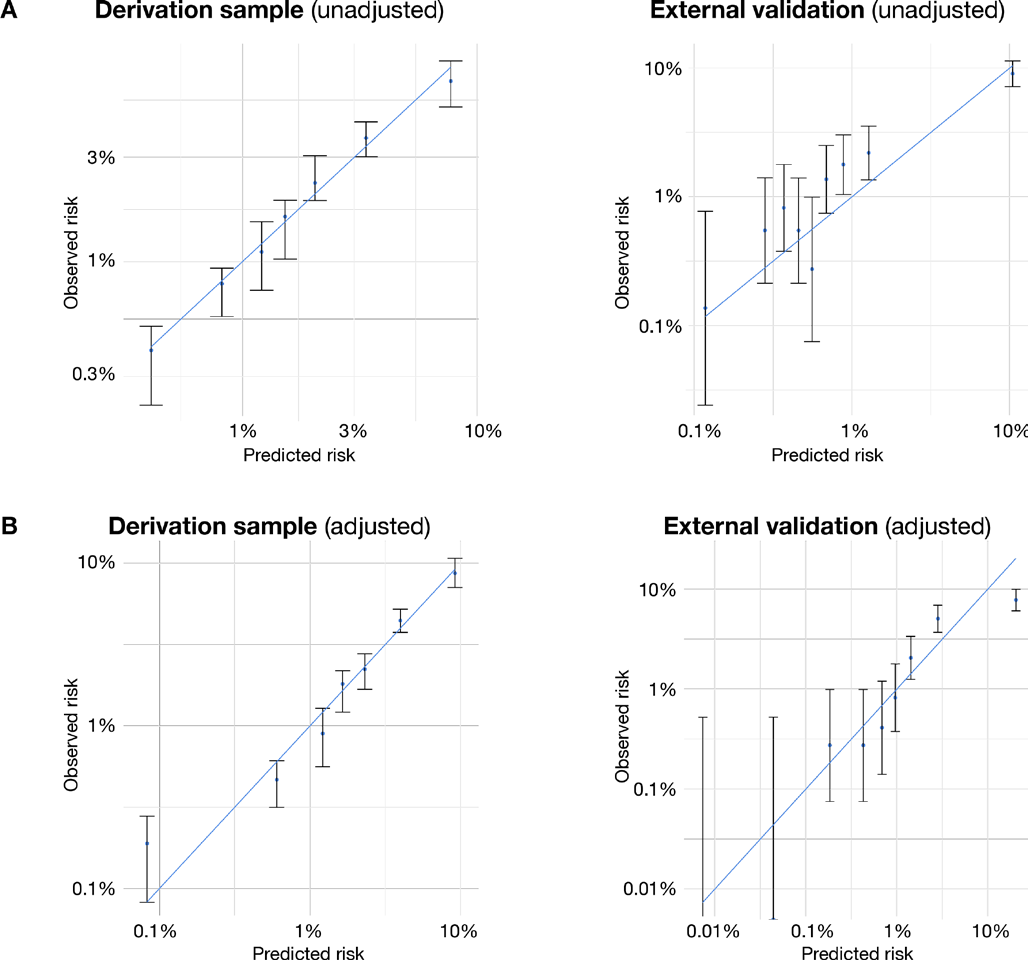
Calibration plots comparing the predicted and observed risks of hospitalisation using (A) the unadjusted model and (B) the model adjusted for interorganisation differences in hospitalisation propensity. Error bars represent 95% CIs.

The clinical prediction models showed good overall discrimination in both internal validation (c-index 0.74, 95% CI 0.72 to 0.76 for the unadjusted model and 0.80, 95% CI 0.78 to 0.82 for the adjusted model) and external validation (c-index 0.80, 95% CI 0.78 to 0.82 for the unadjusted model and 0.84, 95% CI 0.82 to 0.86 for the adjusted model). Figure 1A, B shows receiver operating characteristics, and online supplemental table 4 provides discrimination statistics for both the unadjusted and adjusted models. The adjusted model achieved an NPV of 98.8% and 98.3% in the internal and external validation, respectively, for a PPV of 9.8% and 11.1%, respectively. Calibration plots indicate adequate calibration of the predicted probabilities against observed proportions of psychiatric hospitalisation in both internal and external validation (figure 2A, B). The Brier scores were 0.013 in the internal validation and 0.018 in the external validation, for both the unadjusted and adjusted models.

Both the unadjusted and adjusted predictive models had significantly greater discrimination power than the baseline model (mean gain in c-index for the unadjusted model: 0.13, 95% CI 0.10 to 0.17, p<0.001; and for the adjusted model: 0.18, 95% CI 0.14 to 0.23, p<0.001) and the clinical benchmark model (mean gain in c-index for the unadjusted model: 0.11, 95% CI 0.076 to 0.14, p<0.001; and for the adjusted model: 0.15, 95% CI 0.11 to 0.20, p<0.001) as shown in online supplemental table 4 and figure 1.

**Figure 3:**
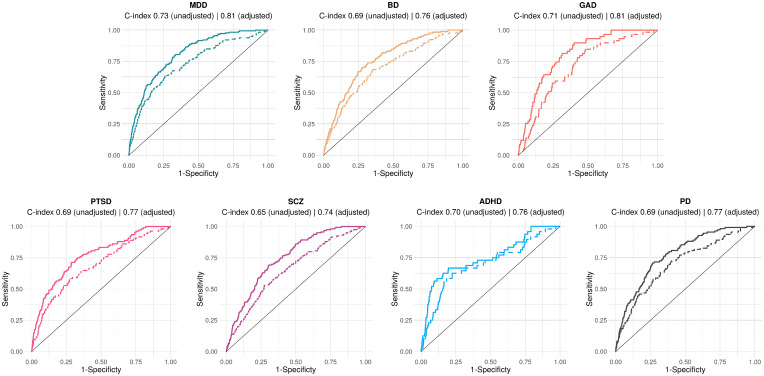
Receiver operating characteristics for the clinical prediction model in each diagnostic category, adjusted (solid line) and unadjusted (dashed line) for interorganisation differences in hospitalisation propensity. Optimism-adjusted c-indices are provided for each diagnostic category. ADHD, attention deficit hyperactivity disorder; BD, bipolar disorder; GAD, generalised anxiety disorder; MDD, major depressive disorder; PD, personality disorder; PTSD, post-traumatic stress disorder; SCZ, schizophrenia or schizoaffective disorder.

The models were found to have good discrimination across all diagnostic categories, especially when adjusting for interorganisation differences in hospitalisation propensity (figure 3), with c-indices ranging from 0.65 (for SCZ) to 0.73 (for MDD) without adjustment and from 0.74 (for SCZ) to 0.81 (for MDD and GAD) with adjustment. No significant difference in c-index was observed between white and non-white people: c-index for the unadjusted model 0.72 for white and 0.75 for non-white people (permutation test p=0.10) and c-index for the adjusted model 0.79 for white and 0.81 for non-white people (p=0.41).

## Discussion

We have developed a prediction model for the 6-month risk of first psychiatric hospitalisation based on longitudinal measurements of two simple clinical scales (CGI-S and GAF). This model had good discrimination and calibration properties in both our internal sample and in a separate sample from other healthcare organisations. It also has adequate discriminative power across psychiatric diagnoses, and its performance does not clearly differ between white and non-white people.

Besides a few readily available factors (age, gender and diagnosis), the predictive model only relies on repeated measurements of 1-item clinical scales (CGI-S and GAF) that do not add a significant burden to routine clinical care—thus overcoming a critical barrier to the adoption of measurement-based care.^25^ Indeed, all measurements used in this study were collected by clinicians as part of routine care and without specific training. From these repeated measurements, clinical severity and instability and functional severity and instability can be automatically calculated. These form the building blocks of the model, akin to the vitals included in early warning scores used in physical health. As such, we see the proposed prediction model as an early warning score for psychiatric hospitalisation. In practice, once implemented, the early warning score would automatically compute and update predictions whenever clinicians input CGI-S and GAF measurements for their patients. The predicted probability of hospitalisation would then be displayed directly to the clinician to inform clinical care. Like any early warning score, it should be used as an evidence-based adjunct to clinical decision-making which will need to be complemented by individual and contextual factors.

Several key steps are required before the early warning score can be implemented in clinical practice. First, prospective validation is needed in individuals who would not typically have five or more CGI-S and GAF measurements recorded within a 6-month period. The current model cannot be assumed to generalise to these individuals. Although the selected cohort was broadly similar to the wider population (online supplemental table 1), differences in racial composition and diagnostic distribution were noted, and unmeasured differences may also exist. Likewise, external validation in healthcare organisations outside the NeuroBlu network, as well as prospective validation in centres that do not currently measure CGI-S and GAF routinely, is also needed. Second, the clinical utility of the model should be evaluated in the context of available interventions. For example, the score could ultimately inform referrals to crisis resolution and home treatment teams (or equivalent more intensive services in other jurisdictions). It will be important to assess whether the score effectively identifies patients most likely to benefit from such interventions. Third, the early warning score will need to be translated into a practical tool for clinicians (whether integrated into EHR systems or delivered through a standalone application) and will require appropriate regulatory approval. Finally, a net benefit analysis and health economic evaluation will be necessary to determine the cost-effectiveness and broader impact of implementing the score in routine care.

When predicting rare but serious events such as psychiatric hospitalisation, the goal is to stratify individuals according to their risk so that effective interventions can be targeted to at-risk individuals. The predicted risk need not be very high to inform clinical decision-making. As an illustration (see online supplemental material I), consider a population of 20 000 individuals with a 2% 6-month risk of psychiatric hospitalisation. Individuals can be referred to a crisis resolution team—an intervention that reduces the odds of psychiatric hospitalisation within the next 6 months by 80%^8^ when implemented with high fidelity.^26^ If it is applied to 1000 individuals selected based on a system with a PPV of 5.4% (corresponding to the clinical benchmark model), 43 hospitalisations would be prevented. But if applied to 1000 individuals selected based on the proposed prediction model (with a PPV of 9.8%), 77 hospitalisations would be prevented and to prevent 43 hospitalisations, the intervention would only need to be applied to 560 individuals (ie, a 44% saving without deploying any additional resources). By way of comparison, the PPV and NPV of our model are similar to those of the first version of the QRISK score that is used across the National Health Service to predict cardiovascular diseases.^27^ Predictive models with PPV of about 10% or less are commonplace in clinical medicine, as in the recommendation to start statins in people with a 10% predicted risk of cardiovascular event^28^ or the recommendation to give tamoxifen to all women with a 3% predicted risk of breast cancer.^29^ This is especially the case when an effective and acceptable intervention (such as crisis resolution team) exists to mitigate the risk of a severe outcome (such as psychiatric hospitalisation). Because false negatives (ie, missing someone at risk of hospitalisation) might come at a high cost for the patient and healthcare provider, the high NPV (98.8%) is particularly important. Given that the early warning score is well calibrated, the cut-off can be adapted to the application, depending on the consequences of false negatives and the nature of interventions that can mitigate the risks.

The science of psychiatric hospitalisation prediction has primarily focused on identifying significant predictors rather than developing clinical prediction models.^10^,^1216^ The few existing models have been limited to specific clinical scenarios (eg, first episode of psychosis^13^ or patients in crisis^14^) and were either not externally validated^14 15^ or achieved limited discrimination in external samples.^13^ In addition, they considered both first and recurrent hospitalisations. Predicting first hospitalisation is more challenging (because prior hospitalisation is an excellent predictor of future hospitalisations^30^) but also more clinically useful (because clinicians cannot rely on past admission patterns in an individual who has never been hospitalised).

By contrast, the transdiagnostic validity of the proposed early warning score to predict first hospitalisation means that it can be used across psychiatric disciplines, when diagnostic uncertainty is present, and in individuals who have not been previously hospitalised. The external validity of the prediction model in patients unseen during its derivation is critical to support its generalisability.

While the early warning score could discriminate between those most and those least at risk of hospitalisation across diagnoses, its performance was better for MDD and GAD than for SCZ (figure 3). This might be because a diagnosis of SCZ trumps the other predictors in forecasting hospitalisation, and instability is a weaker predictor of hospitalisation for SCZ.^16^ The performance of the early warning score was also found to be better in the external validation than in the development sample. This can be explained by a slightly higher rate of psychiatric hospitalisation in the external validation sample and a higher prevalence of MDD (table 1).

This study has several strengths, including a large sample size, prespecified analyses, external validation and transdiagnostic validation. It also has several limitations besides the many steps required before the score can be used in routine clinical care (as discussed above). First, it relies on data from the US, and the early warning score will therefore need to be externally validated in other countries. Second, we limited the investigation to one set of measurements per individual. In practice, the early warning score and estimated risk might be updated dynamically as new measurements accumulate. Whether this provides intra-individual identification of at-risk states remains to be determined. Third, we did not separately assess voluntary and compulsory admissions, and we did not assess the risk of recurrent admissions as explained above. Fourth, while a range of diagnoses were included, others were not (eg, dementia) due to low sample sizes. Fifth, because this study is observational, one cannot infer that reducing the predicted risk will necessarily lower the observed risk of hospitalisation. Sixth, while the adjusted model presented improved discrimination over the unadjusted one, its calibration plot in the external validation shows evidence of overestimation of risk for those with higher hospitalisation risk, suggesting that recalibration of the adjusted model might be needed to optimise its performance in new healthcare organisations. Seventh, while our goal was to develop and validate a model that is applicable in general, further work should examine the model performance in specific situations (eg, in patients in crisis, or in patients seen in specific services).

In summary, easily acquired clinician estimates of overall severity and function can be used in a transdiagnostic early warning score to predict psychiatric hospitalisation and facilitate evidence-based clinical decision-making. This prediction system can help target effective interventions to the patients most likely to benefit from them.

## Supplementary material

10.1136/bmjment-2025-301622 online supplemental file 1

## Data Availability

Data may be obtained from a third party and are not publicly available.
