# Supplementary material for: Transdiagnostic early warning score for psychiatric hospitalisation: development and evaluation of a prediction model
Source: BMJ Ment Health. 2025 Jul 17;28(1):e301622. doi: 10.1136/bmjment-2025-301622 (PMC12273086; doi:10.1136/bmjment-2025-301622)
Supplement: online supplemental file 1 [file bmjment-28-1-s001.pdf]

A transdiagnostic early warning score for psychiatric hospitalisation

Supplemental material

Supplemental A: Statistical analytic plan

Development and validation of an early warning score for psychiatric hospitalisation – 30/01/2024

Table of contents

1. Study objective 2
2. Data 2
3. Cohort criteria 2
4. Diagnostic codes 2
5. Outcome definition 3
6. Predictors definition 3
7. Definition of the derivation and external validation sample 10
8. Primary analysis 4
   8.1 Additional predictors 4
   8.2 Variable transformation 5
   8.3 Survival analysis 5
   8.4 Internal validation 5
   8.5 External validation 6
9. Secondary analyses 6
   9.1 Transdiagnostic evaluation 6
   9.2 Baseline and clinical benchmark models 6

References 6

1. Study objective

The objective of this study is to assess whether measurements of clinical and functional severity and instability (based on longitudinal measurements of clinical global impression scale (CGI-S) and global assessment of functioning (GAF) over a six-month period) can be combined into a clinical prediction model to predict psychiatric hospitalisation within the next 6 months.

2. Data

All analyses are based on data from the NeuroBlu database release 23R3.

3. Cohort criteria

The cohort includes all participants who meet the following criteria:

A. The individual has at least 5 CGI-S and 5 GAF recorded in any 6-month period. We refer to this 6-month period as the measurement period.

B. The individual has any of the following diagnoses in their health records: attention-deficit hyperactivity disorder (ADHD), bipolar disorder (BD), generalised anxiety disorder (GAD), major depressive disorder (MDD), personality disorder (PD), post-traumatic stress disorder (PTSD), schizophrenia or schizoaffective disorder (SCZ). See ICD codes below.

C. The individual has not been hospitalised before or during the measurement period.

D. The individual receives their mental health care in a healthcare organisation (HCO) of the NeuroBlu network, excluding HCOs that have more than 25% of their patients (among those meeting criteria A, B, and C) being admitted to a psychiatric hospital. This criterion excludes HCOs that predominantly have inpatient facilities (for which patients might receive community care in other organizations not included in the network).

When multiple measurement periods allow an individual to be included in the cohort, the earliest period is selected. This defines the beginning of follow-up.

4. Diagnostic codes

The following diagnostic codes are used.

- Major Depressive Disorder (MDD):
  - ICD-9 codes : '296.20', '296.21', '296.22', '296.23', '296.24', '296.25', '296.26', '296.30', '296.31', '296.32', '296.33', '296.34', '296.35', '296.36'
  - ICD-10 codes : 'F32.0', 'F32.1', 'F32.2', 'F32.3', 'F32.4', 'F32.5', 'F32.81', 'F32.89', 'F32.9', 'F33.0', 'F33.1', 'F33.2', 'F33.3', 'F33.40', 'F33.41', 'F33.42', 'F33.8', 'F33.9'
- Bipolar disorder (BD):
  - ICD-9 codes : '296.40', '296.41', '296.42', '296.43', '296.44', '296.45', '296.46', '296.50', '296.51', '296.52', '296.53', '296.54', '296.55', '296.56', '296.60', '296.61', '296.62', '296.63', '296.64', '296.65', '296.66', '296.7', '296.80', '296.89'
  - ICD-10 codes : 'F31.0', 'F31.10', 'F31.11', 'F31.12', 'F31.13', 'F31.2', 'F31.30', 'F31.31', 'F31.32', 'F31.4', 'F31.5', 'F31.60', 'F31.61', 'F31.62', 'F31.63', 'F31.64',

'F31.70', 'F31.71', 'F31.72', 'F31.73', 'F31.74', 'F31.75', 'F31.76', 'F31.77', 'F31.78', 'F31.81', 'F31.89', 'F31.9'

- Generalised Anxiety Disorder (GAD):
  - ICD-9 code: '300.02'
  - ICD-10 code: 'F41.1'
- Post-Traumatic Stress Disorder (PTSD):
  - ICD-9 codes: '309.81'
  - ICD-10 codes: 'F43.10', 'F43.11', 'F43.12'
- Schizophrenia/Schizoaffective disorder (SCZ):
  - ICD-9 codes : '295.00', '295.01', '295.02', '295.03', '295.04', '295.05', '295.10', '295.11', '295.12', '295.13', '295.14', '295.15', '295.20', '295.21', '295.22', '295.23', '295.24', '295.25', '295.30', '295.31', '295.32', '295.33', '295.34', '295.35', '295.40', '295.41', '295.42', '295.43', '295.44', '295.45', '295.50', '295.51', '295.52', '295.53', '295.54', '295.55', '295.60', '295.61', '295.62', '295.63', '295.64', '295.65', '295.80', '295.81', '295.82', '295.83', '295.84', '295.85', '295.90', '295.91', '295.92', '295.93', '295.94', '295.95', '295.7', '295.7x'
  - ICD-10 codes : 'F20.0', 'F20.1', 'F20.2', 'F20.3', 'F20.5', 'F20.81', 'F20.89', 'F20.9', 'F25'
- Attention Deficit Hyperactivity Disorder (ADHD) :
  - ICD-9 codes : '314.00', '314.01'
  - ICD-10 codes : 'F90.0', 'F90.1', 'F90.2', 'F90.8', 'F90.9'
- Personality Disorder (PD) :
  - ICD-9 codes : '301.0', '301.10', '301.11', '301.12', '301.13', '301.20', '301.21', '301.22', '301.3', '301.4', '301.50', '301.51', '301.59', '301.6', '301.7', '301.81', '301.82', '301.83', '301.84', '301.89', '301.9'
  - ICD-10 codes : 'F60.0', 'F60.1', 'F60.2', 'F60.3', 'F60.4', 'F60.5', 'F60.6', 'F60.7', 'F60.81', 'F60.89', 'F60.9'

5. Outcome definition

The primary outcome is admission to a psychiatric hospital as coded using the Observational Medical Outcomes Partnership Common Data Model (inpatient stay)[1] within 180 days after the end of the measurement period. The period of 180 days for follow-up was chosen as it corresponds to the period during which crisis resolution intervention is effective at preventing admission to a psychiatric hospital.[2]

6. Predictors definition

For each individual, clinical severity is defined as the average value of CGI-S in the measurement period:

$$\text{Clinical severity} = \frac{1}{N}\sum_{i=1}^{N} C_i,$$

where C_i is the i-th measurement of C-GIS and N (≥ 5) is the total number of measurements available during the measurement period. Clinical instability is defined as the time-adjusted root mean square of successive differences[3]:

$$\text{Clinical instability} = \sqrt{\frac{1}{N}\sum_{i=1}^{N-1}\left(\frac{C_{i+1}-C_i}{t_{i+1}-t_i}\right)^2},$$

where t_i is the time (timestamp) of the i-th measurement. This can be simply calculated using the following code in R:

```
tRMSSD <- function(times,values){
                n=length(values)
                dtime = times[2:n]-times[1:n-1]
                dvals = values[2:n]-values[1:n-1]
                res = sqrt(1/n*sum((dvals/dtime)^2))
                return(res)

}
```

Similarly, functional severity and instability can be simply calculated as follows:

$$\text{Functional severity} = \frac{1}{M}\sum_{i=1}^{M} G_i,$$

$$\text{Functional instability} = \sqrt{\frac{1}{M}\sum_{i=1}^{M-1}\left(\frac{G_{i+1}-G_i}{t_{i+1}-t_i}\right)^2},$$

where G_i is the i-th value of GAF recorded during the measurement period and M (≥ 5) is the total number of measurements of GAF available during the measurement period.

For each individual in the cohort, we therefore have 4 metrics capturing different aspects of their illness trajectory during the measurement period: clinical severity, clinical instability, functional severity, and functional instability. These are the predictors that will be used in the clinical prediction model.

7. Definition of the derivation and external validation sample

Individuals are separated into a derivation sample and an external validation sample based on the HCOs within which they received their mental health care. This is achieved as follows.

1. For each HCO, the mean date of the beginning of the measurement window for all individuals receiving their care at that organisation is recorded.
2. HCOs are ranked from those providing the most recent data to those providing the least recent data.
3. HCOs are split between a derivation and an external validation sample such that the derivation sample contains the most recent data and the external sample contains the least recent data, and so that the split leads to a ratio that is as close as possible to 80% of individuals in the derivation sample and 20% of individuals in the external validation sample.

8. Primary analysis

8.1 Additional predictors

In the primary analysis, we will build two clinical prediction models. Both include the four predictors described above as well as the following additional predictors:

- Gender: a categorical variable with 3 possible values (Female, Male, Other/Unknown)
- Age: a continuous variable, standardised
- Diagnosis: each of the diagnostic categories defined above, each represented by a binary variable set to 'true' if the individual had that diagnosis recorded at any point during or before the measurement period and 'false' otherwise. This encoding accommodates individuals who have more than one diagnosis.

These additional predictors were selected as they are readily available in clinical practice and are likely associated with likelihood of psychiatric hospitalisation.

In one of the two models, we also include an HCO-wide predictor representing the propensity (logit of the probability) for an individual treated at that HCO to be admitted to a psychiatric hospital. This enables to adjust for differences between HCOs in terms of their hospitalisation policies/practices. It is anticipated that adjusting for this factor will increase the predictive power of the model. However, both models are kept and evaluated separately since there can be contexts wherein the HCO-wide hospitalisation propensity is unavailable (e.g. for a new HCO or when an HCO does not want to share such data or wants to use the early warning system on a subgroup of their patients only).

8.2 Variable transformation

Based on our previous research,[3] we know that the distributions of instability measurements can have a heavy tail. Variable transformation might help stabilise model fitting. Because instability can be equal to zero, variable transformation needs to be defined at zero (thus excluding functions such as the log-transform). We will test a simple square-root transformation of instabilities, and select this variable transformation over none if it leads to a lower Akaike Information Criterion (AIC) for the model.

8.3 Survival analysis

The predictors described above will all be included into a Cox proportional hazard model to predict the outcome. Individuals who a not hospitalised during the follow-up will be censored at their last visit to an HCO or at the end of follow-up (180 days) whichever came first.

The proportional hazard assumption will be tested with the generalised Schoenfeld approach[4] and rejected if p is less than 0.05. If the assumption is violated, then time-varying hazard ratio will be calculated with natural cubic splines fitted to the log cumulative hazard.[5] This is achieved using the generalized survival models of the rstpm2 package (version 1.5.1) in R. As recommended by Royston and Parmar, [5] splines with 1, 2, and 3 degrees of freedom will be estimated for both the baseline log-cumulative hazard and its cohort dependency and the number of degrees of freedom leading to the lowest Akaike Information Criterion (AIC) will be selected.

8.4 Internal validation

In internal validation, discrimination of the model will be assessed by computing the optimism-corrected c-index (using bootstrap with 200 repetitions),[6] as well as sensitivity, specificity, positive predictive value (PPV), and negative predictive value (NPV) for an estimated incidence of hospitalization of 2%.

Confidence intervals for all these quantities will be calculated using bootstrap with 200 repetitions. To avoid nested bootstrap (which leads to prohibitive computational time) which would arise from bootstrapping within each bootstrap iteration to correct for optimism, the optimism-correction from the point estimate will be transported to the confidence interval (i.e. the same adjustment as for the point estimate will be applied to each bootstrap estimate). Calibration will be assessed by plotting the predicted risks against the observed risks, with confidence intervals established using bootstrap with 200 repetitions.[7]

8.5 External validation

To assess the external validity of the predictive model, the same discrimination statistics listed for the internal validation and the relation between the predicted and observed risks will be calculated in the external validation sample, based on the model fitted on the derivation sample. Bootstrap with 1000 repetitions will be used to calculate confidence intervals for the c-index. The reason for the increased number of bootstrap samples is that the sample size is smaller making both the computation time shorter and the uncertainty larger. Wilson score confidence intervals will be calculated for the observed risks. This confidence interval has coverage probability that is closer to the nominal value (95%) even for small number of events, unlike Wald and "exact" confidence intervals.[8]

9. Secondary analyses

9.1 Transdiagnostic evaluation

To assess the transdiagnostic validity of the prediction model, receiver operating characteristics and c-index will be computed after applying the model to each subgroup of individuals defined based on individual diagnostic categories.

9.2 Baseline and clinical benchmark models

To help interpret the performance of the mode, we will compare it to two other models:

- A baseline model that only includes the additional predictors described in Section 8.1. This model does not include any metrics based on clinical and functional measurements.
- A clinical benchmark model that includes all additional predictors described in Section 8.1 and the clinical severity described in Section 6. This is thought to mimic the information that clinicians use to make decisions about patients likely to need hospitalisation.

All analyses presented in Section 8 will be repeated with these two models.

References

1 Patel R, Wee SN, Ramaswamy R, *et al.* NeuroBlu, an electronic health record (EHR) trusted research environment (TRE) to support mental healthcare analytics with real-world data. *BMJ Open* 2022; **12**: e057227.

2 Johnson S, Nolan F, Pilling S, *et al.* Randomised controlled trial of acute mental health care by a crisis resolution team: the north Islington crisis study. *BMJ* 2005; **331**: 599.

3 Taquet M, Griffiths K, Palmer EOC, *et al.* Early trajectory of clinical global impression as a transdiagnostic predictor of psychiatric hospitalisation: a retrospective cohort study. *Lancet Psychiatry* 2023; **10**: 334–41.

4 Grambsch PM, Therneau TM. Proportional hazards tests and diagnostics based on weighted residuals. *Biometrika* 1994; **81**: 515.

5 Royston P, Parmar MKB. Flexible parametric proportional-hazards and proportional-odds models for censored survival data, with application to prognostic modelling and estimation of treatment effects. Statistics in Medicine. 2002; **21**: 2175–97.

6 Harrell FE. Evaluating the yield of medical tests. *JAMA* 1982; **247**: 2543–6.

7 Harrell FE Jr, Lee KL, Mark DB. Multivariable prognostic models: issues in developing models, evaluating assumptions and adequacy, and measuring and reducing errors. *Stat Med* 1996; **15**: 361–87.

8 Agresti A, Coull BA. Approximate is better than "exact" for interval estimation of binomial proportions. *Am Stat* 1998; **52**: 119–26.

Supplemental B: Cohort definition

A. The cohort included all participants who met the following criteria:
B. The individual had at least 5 CGI-S and 5 GAF recorded in any 6-month period. We refer to this 6-month period as the measurement period.
C. The individual had any of the following diagnoses in their health records: attention-deficit hyperactivity disorder (ADHD), bipolar disorder (BD), generalised anxiety disorder (GAD), major depressive disorder (MDD), personality disorder (PD), post-traumatic stress disorder (PTSD), schizophrenia or schizoaffective disorder (SCZ). See ICD codes below.
D. The individual did not have any psychiatric hospitalization recorded before or during the measurement period.
E. The individual received their mental health care in a healthcare organisation (HCO) of the NeuroBlu network, excluding HCOs that have more than 25% of their patients (among those meeting criteria A, B, and C) being admitted to a psychiatric hospital. This criterion excludes HCOs that predominantly have inpatient facilities (for which patients might receive community care in other organizations not included in the network).

When multiple measurement periods allow an individual to be included in the cohort, the earliest period was selected. This defines the beginning of follow-up.

Supplemental C: Diagnostic codes

The following diagnostic codes were used to define diagnoses in the NeuroBlu data.

- Attention Deficit Hyperactivity Disorder (ADHD):
    - ICD-9 codes : '314.00', '314.01'
    - ICD-10 codes : 'F90.0', 'F90.1', 'F90.2', 'F90.8', 'F90.9'
- Bipolar disorder (BD):

- ICD-9 codes : '296.40', '296.41', '296.42', '296.43', '296.44', '296.45', '296.46', '296.50', '296.51', '296.52', '296.53', '296.54', '296.55', '296.56', '296.60', '296.61', '296.62', '296.63', '296.64', '296.65', '296.66', '296.7', '296.80', '296.89'
- ICD-10 codes : 'F31.0', 'F31.10', 'F31.11', 'F31.12', 'F31.13', 'F31.2', 'F31.30', 'F31.31', 'F31.32', 'F31.4', 'F31.5', 'F31.60', 'F31.61', 'F31.62', 'F31.63', 'F31.64', 'F31.70', 'F31.71', 'F31.72', 'F31.73', 'F31.74', 'F31.75', 'F31.76', 'F31.77', 'F31.78', 'F31.81', 'F31.89', 'F31.9'

- Generalised Anxiety Disorder (GAD):
  - ICD-9 code: '300.02'
  - ICD-10 code: 'F41.1'
- Major Depressive Disorder (MDD):
  - ICD-9 codes : '296.20', '296.21', '296.22', '296.23', '296.24', '296.25', '296.26', '296.30', '296.31', '296.32', '296.33', '296.34', '296.35', '296.36'
  - ICD-10 codes : 'F32.0', 'F32.1', 'F32.2', 'F32.3', 'F32.4', 'F32.5', 'F32.81', 'F32.89', 'F32.9', 'F33.0', 'F33.1', 'F33.2', 'F33.3', 'F33.40', 'F33.41', 'F33.42', 'F33.8', 'F33.9'
- Personality Disorder (PD):
  - ICD-9 codes : '301.0', '301.10', '301.11', '301.12', '301.13', '301.20', '301.21', '301.22', '301.3', '301.4', '301.50', '301.51', '301.59', '301.6', '301.7', '301.81', '301.82', '301.83', '301.84', '301.89', '301.9'
  - ICD-10 codes : 'F60.0', 'F60.1', 'F60.2', 'F60.3', 'F60.4', 'F60.5', 'F60.6', 'F60.7', 'F60.81', 'F60.89', 'F60.9'
- Post-Traumatic Stress Disorder (PTSD):
  - ICD-9 codes: '309.81'
  - ICD-10 codes: 'F43.10', 'F43.11', 'F43.12'
- Schizophrenia/Schizoaffective disorder (SCZ):
  - ICD-9 codes : '295.00', '295.01', '295.02', '295.03', '295.04', '295.05', '295.10', '295.11', '295.12', '295.13', '295.14', '295.15', '295.20', '295.21', '295.22', '295.23',

'295.24', '295.25', '295.30', '295.31', '295.32', '295.33', '295.34', '295.35', '295.40', '295.41', '295.42', '295.43', '295.44', '295.45', '295.50', '295.51', '295.52', '295.53', '295.54', '295.55', '295.60', '295.61', '295.62', '295.63', '295.64', '295.65', '295.80', '295.81', '295.82', '295.83', '295.84', '295.85', '295.90', '295.91', '295.92', '295.93', '295.94', '295.95', '295.7', '295.7x'

- ICD-10 codes : 'F20.0', 'F20.1', 'F20.2', 'F20.3', 'F20.5', 'F20.81', 'F20.89', 'F20.9', 'F25'

Supplemental D: Definition of the derivation and external validation sample

Individuals were separated into a derivation sample and an external validation sample based on the HCOs within which they received their mental health care. This was achieved as follows.

For each HCO, the mean date of the beginning of the measurement window for all individuals receiving their care at that organisation was recorded.

HCOs were ranked from those providing the most recent data to those providing the least recent data.

HCOs were split between a derivation and an external validation sample such that the derivation sample contained the most recent data and the external validation sample contained the least recent data, and so that the split led to a ratio that is as close as possible to 80% of individuals in the derivation sample and 20% of individuals in the external validation sample.

Supplemental E: Operationalisation of instability

A full description of clinical instability, the rationale for measuring it, and its operationalisation is provided in a previous study [1].

In short, the instability of a time series is defined as the variability in its values between time points. Applied to a time series of clinical severity (e.g. CGI-S), a patient with a severe disease (high CGI-S) at one visit who then presents with a much less severe illness (low CGI-S) at the next visit, only to

present again with severe illness at the next, would have a high clinical instability. By contrast, a patient with constant clinical severity (whether high or low) would have a low instability.

Instability should not be confounded with variance. The latter does not consider the visit-to-visit variability but instead measures the variability across all time points at once. As such, a patient who starts with a very severe illness and progressively but consistently gets better (going from high CGI-S to a low CGI-S) would have a high variance in CGI-S but a relatively low instability (since only small changes occur between time points).

The mathematical operationalisation of the definition above is:

$$\mathrm{tRMSSD} = \sqrt{\frac{1}{N}\sum_{i=1}^{N-1}\left(\frac{C_{i+1}-C_i}{t_{i+1}-t_i}\right)^2},$$

where C_i is the i-th value of the time series and t_i the time at which the value C_i was recorded. This can be understood as follows. The formula in brackets represents the rate of change in the value C_i between two time points. It is squared because we are interested in the magnitude of the change and not its sign. It is then averaged over all pairs of consecutive values. The square root is then taken to form the root mean square of successive differences.

Supplemental F: Predictor definitions

For each individual, clinical severity was defined as the average value of CGI-S in the measurement period:

$$\text{Clinical severity} = \frac{1}{N}\sum_{i=1}^{N} C_i,$$

where C_i is the i-th measurement of C-GIS and N (≥ 5) is the total number of measurements available during the measurement period. Clinical instability was defined as the time-adjusted root mean square of successive differences:

$$\text{Clinical instability} = \sqrt{\frac{1}{N}\sum_{i=1}^{N-1}\left(\frac{C_{i+1} - C_i}{t_{i+1} - t_i}\right)^2},$$

Similarly, functional severity and instability can be simply calculated as follows:

$$\text{Functional severity} = \frac{1}{M}\sum_{i=1}^{M} G_i,$$

$$\text{Functional instability} = \sqrt{\frac{1}{M}\sum_{i=1}^{M-1}\left(\frac{G_{i+1} - G_i}{t_{i+1} - t_i}\right)^2},$$

where G_i is the i-th value of GAF recorded during the measurement period and M (≥ 5) is the total number of measurements of GAF available during the measurement period.

For each individual in the cohort, we therefore have four metrics capturing different aspects of their illness trajectory during the measurement period: clinical severity, clinical instability, functional severity, and functional instability. These were the predictors that were used in the clinical prediction model.

Based on our previous research [1], we know that the distribution of instability measurements can have a heavy tail. Variable transformation was therefore considered. Because instability can be equal to zero, variable transformation needs to be defined at zero (thus excluding functions such as the log-transform). We therefore tested a square-root transformation of instabilities, and selected this variable transformation over none because it led to a lower Akaike Information Criterion (AIC) for the model.

Supplemental G: Additional predictors

In the primary analysis, we built two clinical prediction models. Both included the four predictors described above as well as the following additional predictors:

Gender: a categorical variable with three possible values (Female, Male, Other/Unknown)

Age: a continuous variable, standardised

Diagnosis: the diagnostic categories defined above, each represented by a binary variable set to 'true' if the individual had that diagnosis recorded at any point during or before the measurement period and 'false' otherwise. This encoding accommodates individuals who have more than one diagnosis.

These additional predictors were selected as they are readily available in clinical practice and are likely associated with the risk of psychiatric hospitalization.

In one of the two models (referred to as the 'adjusted' model), we also included an HCO-wide predictor representing the propensity (logit of the probability) for an individual treated at that HCO to be admitted to a psychiatric hospital. This enables to adjust for differences between HCOs in terms of their hospitalization policies/practices. It is anticipated that adjusting for this factor will increase the predictive power of the model. However, both models are kept and evaluated separately since there can be contexts wherein the HCO-wide hospitalization propensity is unavailable (e.g. for a new HCO or when an HCO does not want to share such data or wants to use the early warning system on a subgroup of their patients only).

Supplemental H: Statistics

The c-index was calculated for the Cox proportional hazard model using the validate function in the rms package, which accounts for censoring.

For sensitivity, specificity, negative and positive predictive values, Brier score, and observed probabilities against which predicted probabilities are compared, censoring of individuals was ignored to determine whether an individual had been hospitalized. For instance, if an individual had their last visit 160 days into the follow-up at which point they were censored and if they had not been hospitalized by that point, then they were considered not to have had the event of interest when calculating summary statistics. This was decided for three reasons. First, no simple formula exists for these quantities that account for censoring. Second, the majority of people were followed up for the whole 180 days (see Table 1). Third, a proportion of people who were "censored" were likely those

who were discharged and did not require hospitalization. These people would therefore be appropriately considered not to have had the event of interest during the follow-up period.

Confidence intervals for the c-index were calculated using bootstrap with 200 repetitions. To avoid nested bootstrap (leading to prohibitive computational time) which would arise from bootstrapping within each bootstrap iteration to correct for optimism, the optimism-correction from the point estimate was transported to the confidence interval (i.e. the same adjustment as for the point estimate was applied to each bootstrap estimate).

Supplemental I: Illustration of the clinical meaningfulness of the model

In the discussion, we translated the positive and negative predictive values of the model into the number of hospitalizations that could be prevented if used alongside an intervention (crisis resolution team). Here we explain how we arrived at these numbers.

For a population of 20,000 patients with a 2% risk of hospitalization without intervention (i.e. 400 hospitalizations in total), if a model has a PPV of 9.8% and an NPV of 98.8%, then the contingency table is as follows:

	**Hospitalized** if not treated	**Non-hospitalized**
**Tested positive**	182	1678
**Tested negative**	218	17922

If we can treat 1000 people, we would select 1000 people randomly from the 1860 (i.e. 182+1678) who tested positive (to maximize the yield of the intervention). We would therefore select on average 98 (1000/1860x182) people from those who would have been hospitalised if not treated and 902

people from those who not have been hospitalized anyway. The odds of being hospitalized in this cohort without any intervention would be 98/902 = 0.109. Since the intervention (crisis resolution team) reduces the odds by 80%, the odds after intervention would be 0.0217 which implies that only 21 people out of the 1000 treated would be hospitalized, and 979 would not. This implies that 77 hospitalizations would be prevented (from 98 to 21).

The same reasoning applied to a model with a PPV of 5.4% would start with the following contingency table:

	**Hospitalized** if not treated	**Non-hospitalized**
**Tested positive**	206	3604
**Tested negative**	194	15996

from which it can be shown that 43 hospitalizations would be prevented by targeting the intervention to those who tested positive.

SUPPLEMENTAL FIGURE

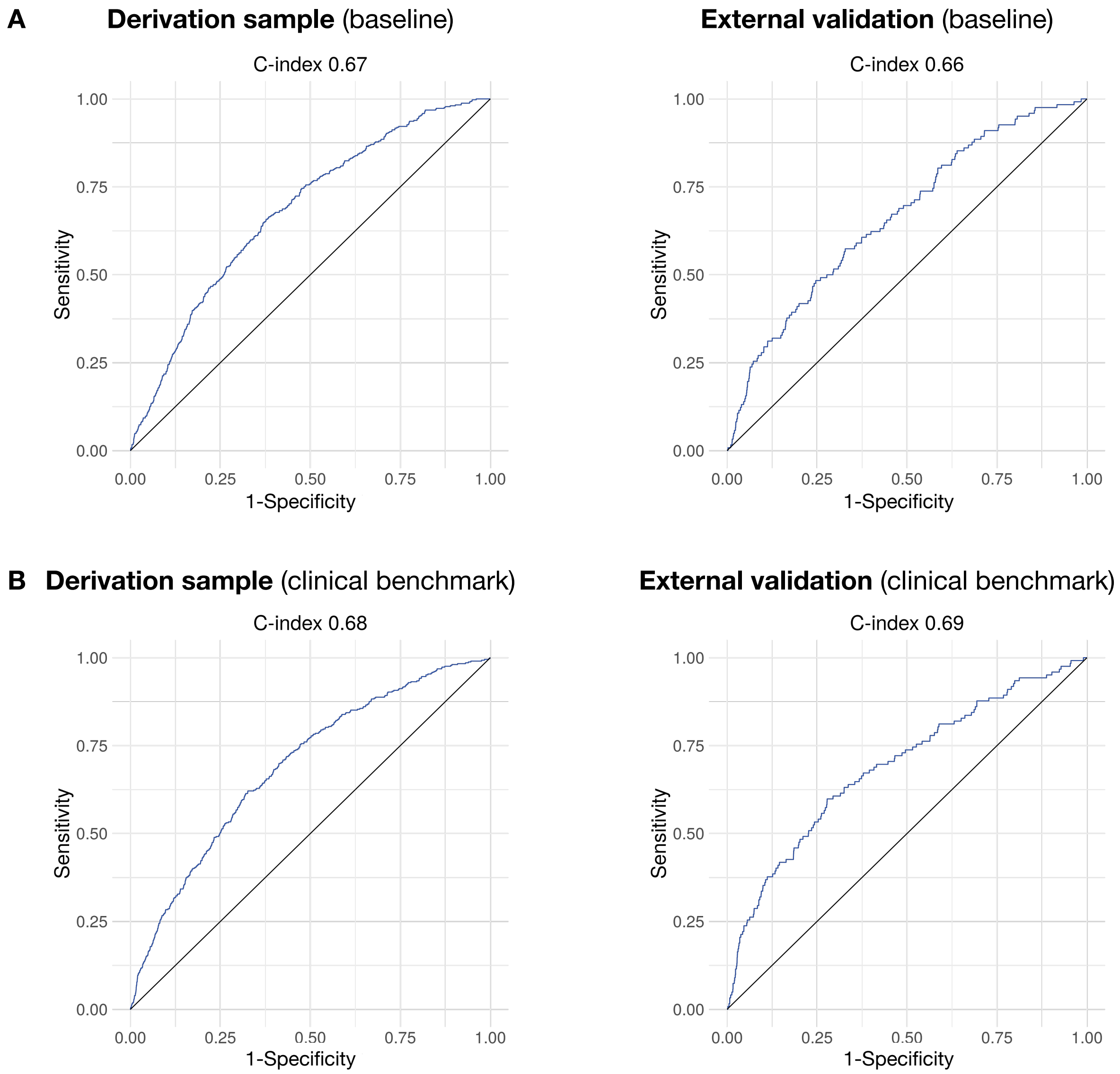

Supplemental Figure 1. Receiver operating characteristics for the prediction of 6-month hospitalization using (A) the baseline model, and (B) the clinical benchmark model.

SUPPLEMENTAL TABLES

Supplemental Table 1. Characteristics of the selected cohort compared to those of the wider population with any number of CGI-S/GAF measurements and without sites being excluded. SMD = Standardised mean difference. Note that unlike in Table 1, the CGI-S and GAF are reported as mean and SD rather than median and IQR as these are the input to calculate the SMD.

	**Cohort 1** - Wider population with any number of CGI-S/GAF measurements and no site excluded	**Cohort 2** - Wider population with any number of CGI-S/GAF measurements	**Cohort 3** - Selected cohort for the primary analysis	**SMD between 1 and 3**	**SMD between 2 and 3**
**Cohort size**	96,715	87,998	37,049	-	-
**Age [years], mean (SD)**	32.06 (18.22)	31.91 (18.31)	32.09 (18.67)	0.0016	0.0098
**Gender, n (%)**					
Female	54848 (56.71)	50397 (57.27)	21335 (57.59)	0.018	0.0065
Male	41836 (43.26)	37574 (42.70)	15706 (42.39)	0.018	0.0063
Unknown	31 (0.032)	27 (0.031)	8 (0.022)	0.0061	0.0055
**Race, n (%)**					
American Indian or Alaska Native	392 (0.41)	384 (0.44)	146 (0.39)	0.0032	0.0078
Asian	705 (0.73)	630 (0.72)	302 (0.82)	0.01	0.011
Black or African American	16043 (16.59)	14352 (16.31)	7183 (19.39)	0.073	0.08
Native Hawaiian or Other Pacific Islander	917 (0.95)	88 (0.10)	43 (0.12)	0.11	0.006
No matching concept	22236 (22.99)	21326 (24.23)	9824 (26.52)	0.082	0.053
White	56422 (58.34)	51218 (58.20)	19551 (52.77)	0.11	0.11
**Diagnosis, n (%)**					
MDD	34260 (35.42)	30870 (35.08)	13503 (36.45)	0.021	0.029
BD	20450 (21.14)	17856 (20.29)	8271 (22.32)	0.029	0.05
GAD	12261 (12.68)	11466 (13.03)	4991 (13.47)	0.023	0.013
PTSD	17938 (18.55)	17076 (19.40)	8036 (21.69)	0.078	0.057
SCZ	9087 (9.40)	7768 (8.83)	4737 (12.79)	0.11	0.13
ADHD	20863 (21.57)	19923 (22.64)	8737 (23.58)	0.048	0.022
PD	10109 (10.45)	9086 (10.33)	5236 (14.13)	0.11	0.12
**Follow-up, days**	180 (180-180)	180 (180-180)	180 (180-180)	0	0
**Mean GAF**	52.34 (11.99)	52.95 (11.69)	52.15 (10.24)	0.016	0.046
**Mean CGI-S**	4.12 (0.96)	4.07 (0.95)	4.15 (0.87)	0.031	0.0084
**Admissions, n (%)**	1570 (1.62)	1073 (1.22)	531 (1.43)	0.016	0.018

Supplemental Table 2. Coefficients of the unadjusted clinical prediction model

	Coefficient	S.E.	Wald Z	p-value
**Clinical severity**	0.1401	0.0506	2.77	0.0056
**Functional severity**	0.3966	0.053	7.49	<0.0001
**Clinical instability**	0.1232	0.0453	2.72	0.0066
**Functional instability**	0.1828	0.038	4.81	<0.0001
**Male gender**	0.1772	0.1047	1.69	0.0907
**Unknown/Other gender**	-4.5237	15.0971	-0.3	0.7645
**Age**	0.1177	0.0608	1.94	0.0529
**MDD**	-0.0744	0.1213	-0.61	0.5394
**ADHD**	-0.7494	0.1949	-3.85	0.0001
**BD**	0.427	0.1122	3.81	0.0001
**SCZ**	0.6507	0.1245	5.23	<0.0001
**PD**	0.23	0.1275	1.8	0.0712
**PTSD**	0.0359	0.124	0.29	0.7722
**GAD**	0.0771	0.1663	0.46	0.6428

Abbreviations: ADHD, attention-deficit hyperactivity disorder; BD, bipolar disorder; GAD, generalized anxiety disorder; PD, personality disorder; PTSD, post-traumatic stress disorder; SCZ, schizophrenia or schizoaffective disorder; SE, standard error.

Note: Age was standardised (i.e., mean-subtracted and divided by the standard deviation), so if x is the age of an individual, the input to the model should be (x-32.09)/18.67. This also applies to clinical severity so that if x is the value of clinical severity, the input value to the model should be (x-4.15)/0.87. Similarly, if x is the value of functional severity, the input value to the model should be the standardised functional severity: (x-52.15)/10.24. For clinical and functional instability, standardisation was applied to the square-root-transformed values so that if x is the original value of clinical instability, the input to the model should be (sqrt(x)-0.28)/0.27, and for functional instability, the input should be (sqrt(x)-0.71)/0.71. Please see the code available at [URL provided upon acceptance] for details.

Supplemental Table 3. Coefficients of the adjusted clinical prediction model

	**Coefficient**	**S.E.**	**Wald Z**	**p-value**
**Clinical severity**	0.0373	0.0526	0.71	0.479
**Functional severity**	0.308	0.0568	5.42	<0.0001
**Clinical instability**	0.2006	0.0456	4.4	<0.0001
**Functional instability**	0.1359	0.0399	3.41	0.0007
**Male gender**	0.2204	0.1049	2.1	0.0357
**Unknown/Other gender**	-5.5048	25.5124	-0.22	0.8292
**Age**	0.1011	0.0609	1.66	0.0973
**MDD**	-0.0738	0.1217	-0.61	0.5442
**ADHD**	-0.8874	0.1968	-4.51	<0.0001
**BD**	0.3717	0.1135	3.27	0.0011
**SCZ**	0.6443	0.1266	5.09	<0.0001
**PD**	0.1933	0.1276	1.52	0.1297
**PTSD**	0.1319	0.1244	1.06	0.2893
**GAD**	-0.0762	0.1667	-0.46	0.6476
**HCO-wide hospitalization propensity**	1.0904	0.0926	11.77	<0.0001

Abbreviations: ADHD, attention-deficit hyperactivity disorder; BD, bipolar disorder; GAD, generalized anxiety disorder; PD, personality disorder; PTSD, post-traumatic stress disorder; SCZ, schizophrenia or schizoaffective disorder; SE, standard error.

Note: Please refer to the legend of Table S1 for details on inputs to the models.

Supplemental Table 4. Performance of the predictive models (adjusted and unadjusted for organization-level propensity of hospitalization) compared to the baseline and clinical benchmark models.

	C-index	Specificity	Sensitivity	NPV	PPV
**Adjusted model (internal)**	0.80 (0.78-0.82)	98.2 (98.1-98.2)	14.6 (11.1-18.1)	98.8 (98.7-99.0)	9.8 (7.2-12.3)
**Unadjusted model (internal)**	0.74 (0.72-0.76)	98.1 (98.1-98.2)	10.6 (7.5-13.9)	98.8 (98.7-98.9)	7.1 (4.8- 9.7)
**Clinical benchmark model (internal)**	0.68 (0.66-0.71)	98.1 (98.0-98.1)	8.1 (4.6-11.4)	98.7 (98.6-98.9)	5.4 (3.1-7.7)
**Baseline model (internal)**	0.67 (0.65-0.69)	98.1 (98.0-98.1)	6.1 (3.9-8.3)	98.7 (98.6-98.8)	4.1 (2.6-5.6)
**Adjusted model (external)**	0.84 (0.82-0.86)	98.2 (98.1-98.3)	11.9 (6.1-18.2)	98.3 (98.0-98.6)	11.1 (5.4-17.4)
**Unadjusted model (external)**	0.80 (0.78-0.82)	98.1 (98.0-98.3)	10.2 (5.2-15.4)	98.3 (98.0-98.6)	9.5 (4.5-15.2)
**Clinical benchmark model (external)**	0.69 (0.67-0.72)	98.1 (98.0-98.2)	7.9 (3.5-13.0)	98.2 (97.9-98.6)	7.3 (3.0-12.1)
**Baseline model (external)**	0.66 (0.61-0.71)	98.0 (98.0-98.1)	2.9 (0.0-6.7)	98.2 (97.8-98.5)	2.7 (0.0-6.1)

Abbreviations: NPV, negative predictive value; PPV, positive predictive value.

Notes: Performances are shown for both the internal validation (using bootstrapping) and the external validation. Numbers in brackets indicate 95% confidence intervals. The baseline model includes age, gender, and diagnoses as predictors. The clinical benchmark model also includes clinical severity. The unadjusted model adds clinical instability, and functional severity and instability. The adjusted model includes all these predictors as well as a healthcare organization-wide variable representing their propensity to hospitalize patients.

REFERENCES

1 Taquet M, Griffiths K, Palmer EOC, *et al.* Early trajectory of clinical global impression as a transdiagnostic predictor of psychiatric hospitalisation: a retrospective cohort study. *Lancet Psychiatry*. 2023;10:334–41.
